# Generation and characterization of a stable cell population releasing fluorescent HIV-1-based Virus Like Particles in an inducible way

**DOI:** 10.1186/1472-6750-6-52

**Published:** 2006-12-27

**Authors:** Claudia Muratori, Paola D'Aloja, Fabiana Superti, Antonella Tinari, Nathalie Sol-Foulon, Sandra Sparacio, Valerie Bosch, Olivier Schwartz, Maurizio Federico

**Affiliations:** 1 National AIDS Center, Istituto Superiore di Sanità, Viale Regina Elena, 299, 00161 Rome, Italy; 2 Department of Technology and Health, Istituto Superiore di Sanità, Viale Regina Elena, 299, 00161 Rome, Italy; 3 Virus and Immunity Group, Department of Virology, Institut Pasteur, 28 rue du Dr Roux, 75724 Paris Cedex 15, France; 4 Department for Molecular Virology, University of Heidelberg, Otto-Meyerhof-Zentrum, Im Neuenheimer Feld 350, 69120 Heidelberg, Germany; 5 Forschungsschwerpunkt Infektion und Krebs, F020, Deutsches Krebsforschungszentrum, Im Neuenheimer Feld 242, 69120 Heidelberg, Germany

## Abstract

**Background:**

The availability of cell lines releasing fluorescent viral particles can significantly support a variety of investigations, including the study of virus-cell interaction and the screening of antiviral compounds. Regarding HIV-1, the recovery of such biologic reagents represents a very hard challenge due to the intrinsic cytotoxicity of many HIV-1 products. We sought to overcome such a limitation by using a cell line releasing HIV-1 particles in an inducible way, and by exploiting the ability of a HIV-1 Nef mutant to be incorporated in virions at quite high levels.

**Results:**

Here, we report the isolation and characterization of a HIV-1 packaging cell line, termed 18-4s, able to release valuable amounts of fluorescent HIV-1 based Virus-Like Particles (VLPs) in an inducible way. 18-4s cells were recovered by constitutively expressing the HIV-1 NefG3C mutant fused with the enhanced-green fluorescent protein (NefG3C-GFP) in a previously isolated inducible HIV-1 packaging cell line. The G3C mutation creates a palmitoylation site which results in NefG3C-GFP incorporation into virions greatly exceeding that of the wild type counterpart. Upon induction of 18-4s cells with ponasterone A and sodium butyrate, up to 4 μg/ml of VLPs, which had incorporated about 150 molecules of NefG3C-GFP per viral particle, were released into the culture supernatant. Due to their intrinsic strong fluorescence, the 18-4s VLPs were easily detectable by a novel cytofluorometric-based assay developed here. The treatment of target cells with fluorescent 18-4 VLPs pseudotyped with different glycoprotein receptors resulted in these becoming fluorescent as early as two hours post-challenge.

**Conclusion:**

We created a stable cell line releasing fluorescent HIV-1 based VLPs upon induction useful for several applications including the study of virus-cell interactions and the screening of antiviral compounds.

## Background

Fluorescent viral particles represent an extremely useful tool for the study of virus-cell interaction. For this reason many efforts have been devoted to the recovery of fluorescent viruses of different species, including herpesviruses [1,2], adenoviruses [3], rhabdoviruses [4], and the African swine fever virus [5]. Concerning HIV, on the one hand, virions which lead to only the infected cell becoming fluorescent were generated by replacing the *nef *gene with the enhanced green fluorescent protein (*gfp*) gene [6] or by inserting a *gfp-ires *cassette upstream to *nef *[7]. On the other hand, HIV particles, which themselves were fluorescent due to the incorporation of a fluorescent fusion protein, were generated by inserting the *gfp *gene between the matrix and capsid Gag sequences [8]. Of note, however, such engineered HIV-1 derivatives, although replication-competent, suffer of limitations in their replication efficiency. As an alternative to insertion of the GFP gene into the viral genome, fluorescent HIV-1 virions were generated by co-expressing a wild-type HIV-1 genome with the GFP-Vpr fusion product [9,10]. In this case, the fluorescent signal is detectable only in the first replication cycle, and persist until it is intracellularly degraded. Up until now, fluorescent viral particles have been generated by transient transfection procedures and cell lines stably releasing such particles have not yet been available.

Similarly to Vpr, the HIV Nef protein is incorporated in HIV-1 particles, even if at lower levels, i.e. approximately 10 molecules per virion [11-13]. The Nef virion incorporation depends on its localization at the cell membrane. This, in turn, depends on its N-terminus myristoylation marked by the presence of a glycine residue at the position 2, as well as on the presence of two clusters of basic amino acids at the N-terminal region, i.e. two lysines at the positions 4 and 7 and four arginines within the region of amino acid 17–22 [14].

HIV is thought to bud at level of the so called "detergent-resistant microdomains" (rafts) [15], that are regions within the cell membranes enriched in cholesterol and sphingolipids. Several authors demonstrated the presence of Nef in rafts [16-19], even if the relative biologic significance has to be clarified yet. In any case, Nef has been found associating poorly (from 2 to 5%) to lipid rafts [20], and this may account for the low amounts of Nef incorporated into the virions. Hence, a more efficient Nef localization at the lipid raft is expected to increase its viral incorporation. This was indeed the case of the HIV-1 Nef double mutant ^V^153^L^, ^E^177^G ^we recently described, whose strong raft localization leads to a virion incorporation about 100-fold more efficient than its wt counterpart [21].

Palmitoylation is a reversible post-translational protein fatty acylation generally occurring on cysteine residues located close to domains interacting with cell membranes. Such a modification regulates the intracellular protein trafficking and the disposition at the cell membrane rafts. In this regard, it was recently demonstrated that the Nef palmitoylation produced by mutating the Gly 3 to Cys increases the raft localization up to 12-fold compared with wt Nef [20,22]. By consequence, it could be anticipated that palmitoylated Nef undergoes virion incorporation with an efficiency significantly higher than the wild-type counterpart.

Here, we report that a fusion protein consisting of palmitoylated Nef mutant and the green-fluorescent protein (NefG3C-GFP) was efficiently incorporated into HIV-1 particles. In order to generate a stable source of fluorescent HIV-1 virus-like particles (VLPs), we have then stably introduced an expression vector for NefG3C-GFP into a previously described inducibly regulated 293-based HIV-1 packaging cell line. Upon induction of these cells (18-4s), high amounts of fluorescent HIV-1-based VLPs were produced which may be employed for both basic research and antiviral screening.

## Results

### NefG3C-GFP is incorporated in HIV-1 virions at high levels

**Figure 1:**
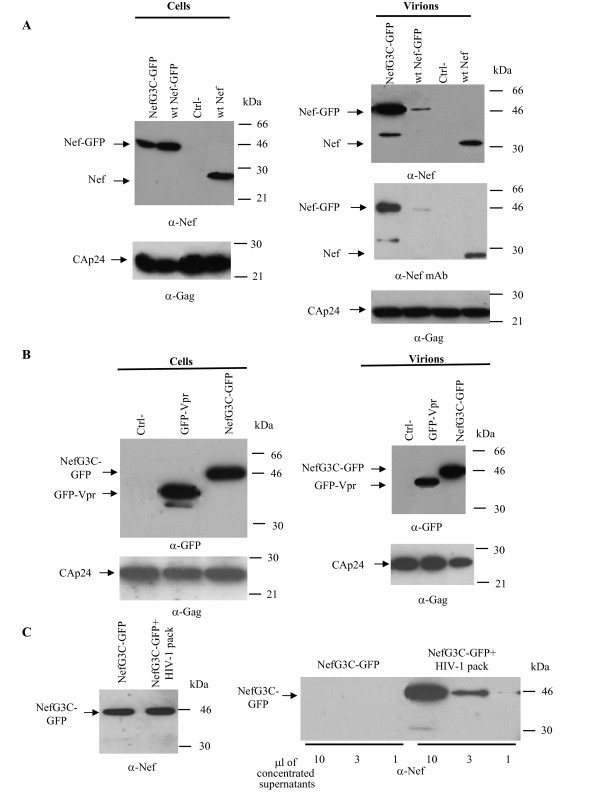
**NefG3C-GFP incorporates at high levels in HIV-1 based VLPs**. Western blot analysis for the virion incorporation of NefG3C-GFP as compared with wt Nef and wt Nef-GFP (A) or with GFP-Vpr (B). 293T cells were transfected with the pCMVΔR8.74 HIV-1 packaging vector together with vectors expressing either wt Nef, wt Nef-GFP, or GFP-Vpr in 1:1 molar ratio. Forty-eight hours later, supernatants were harvested, and the VLPs purified on a 20% sucrose cushion. Then, the VLP concentrations were measured by a quantitative anti-HIV-1 CAp24 ELISA, and 100 ng of VLPs were analyzed by Western blot using a 1:1000 dilution of the ARP444 anti-Nef polyclonal Abs, a 1:200 dilution of the 6.2 anti-Nef mAb (A), or a 1:500 dilution of an anti-GFP mAb (B). As negative control (Ctrl-), 100 ng of empty VLPs were used in both cases. In addition, 50 μg of the lysates from the producer cells were probed for the expression of HIV-1 CAp24, Nef-, and GFP-related products. (C) Anti-Nef Western blot analysis of the indicated volumes of 100-fold concentrated and purified supernatants from 5 × 10^6 ^293T cells transfected with the NefG3C-GFP expression vector alone or together with the pCMVΔR8.74 HIV-1 packaging vector in a 1:1 molar ratio. In the here presented experiment, the virion concentration in the preparation from the cells transfected with the HIV-1 packaging vector was 3 ng/μl of CAp24. Also, 50 μg of the lysates from transfected cells were analyzed for the NefG3C-GFP expression. Data are representative of 9 (A), 3 (B), and 2 (C) independent experiments. The migration of major viral products are indicated on the left side, whereas the molecular marker sizes are reported on the right.

The N-terminal palmitoylation significantly increases the localization at the cell membrane rafts of Nef both in its native form [20] and when fused with GFP [22]. Thus, since rafts are the sites from where HIV preferentially buds [15], we expected that the NefG3C-GFP fusion product would be incorporated into virions at levels which would be high enough to result in the HIV-1 particles being highly fluorescent. To validate such a prediction, we compared the levels of incorporation of NefG3C-GFP into virions with those of wt Nef and wt Nef-GFP. In addition, the virion associated amounts of NefG3C-GFP were compared with those of GFP-Vpr, already known to incorporate efficiently in viral particles [9,10]. We co-transfected 293T cells with the pCMVΔR8.74 HIV-1 packaging construct together with vectors expressing each of the above HIV-1 protein derivatives. Forty-eight hours later, the supernatants were harvested, clarified, and the VLPs purified and analyzed by Western blot for the incorporation of Nef- or GFP-derivatives. We observed that equal amounts of viral particles gave rise to NefG3C-GFP signals much stronger than those produced by both wt Nef and wt Nef-GFP (Figure 1A), and also slightly more intense compared with GFP-Vpr (Figure 1B). This suggests that NefG3C-GFP is incorporated at high levels in HIV-1 viral particles.

Since NefG3C strongly associates with cell membranes [20,22], it is conceivable that at least part of the NefG3C-GFP we detected was associated with microvesicles and/or exosomes rather than to viral particles. To test this possibility, we transfected 293T cells with the NefG3C-GFP expressing vector alone or together with the pCMVΔR8.74 HIV-1 packaging construct. Forty-eight hours later, the supernatants were harvested, concentrated and purified as above described, and equal volumes of each preparation were analyzed by anti-Nef Western blot. As shown in Figure 1C, no detectable amounts of NefG3C-GFP were found in supernatants from 293T cells transfected in the absence of the HIV-1 packaging construct. This strongly suggests that the most part of NefG3C-GFP molecules we detected in supernatants from the cells co-transfected with the HIV-1 packaging construct were indeed associated with viral particles.

These data strongly support the hypothesis that Nef palmitoylation results in a significant increase in virion incorporation levels which was the basis to attempt the generation of cells stably releasing VLPs with incorporated NefG3C-GFP.

### Isolation and induction of the 18-4s cell line stably expressing the NefG3C-GFP fusion product

We have used the 293 Rev-Gag-Pol [23] (henceforth referred to as 293/RGP) as a platform for the generation of stable cells releasing fluorescent HIV-1 based VLPs. 293/RGP cells are packaging cells for HIV-1 derived vectors inducibly expressing and releasing HIV-1 VLPs. In the 293/RGP cells, the HIV-*gag-pol *gene and the HIV-*rev *are separately expressed under the control an ecdysone-inducible promoter (for a review, see [24]) so that particle production requires addition of the ecdysone analogue ponasterone A (PonA) to the medium.

**Figure 2:**
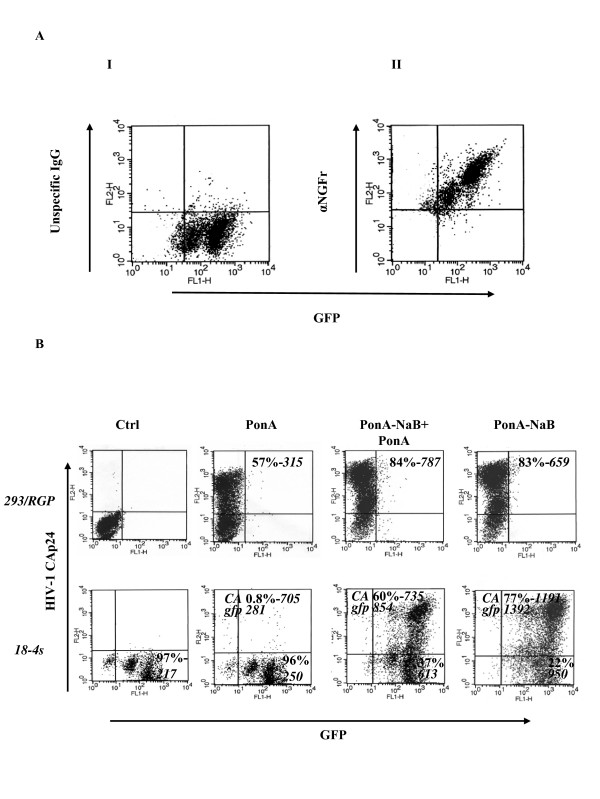
**Analysis of the expression of the transfected vectors in control or induced 18-4s cells**. (A). Analysis of the expression of NefG3C-GFP and ΔNGFr in non induced 18-4s cells. Seven days after the last cell sorter purification step, the cells were labeled with unspecific IgG (I) or with anti-human NGFr mAb (II) and analyzed by FACs. Quadrants were set on the basis of the fluorescence levels of 293/RGP cells labeled with anti-NGFr mAb. (B) FACs analysis for the expression of both NefG3C-GFP and HIV-1 CAp24-related products in 18-4s cells treated with: i) 2 μM PonA; ii) 2 μM PonA and 5 mM sodium butyrate for one day and, thereafter, with 2 μM PonA alone, or iii) 2 μM PonA and 5 mM sodium butyrate. The cells harvested two days after the induction were permeabilized and labeled with a 1:100 dilution of the anti-CAp24 KC-57 PE-conjugated mAb. 293/RGP cells were used as control. Both the percentages and the mean fluorescence values referred to CAp24- and, in the case of 18-4s cells, also to the GFP-related fluorescence are reported in the respective quadrants. NaB: sodium butyrate. The results are representative of 3 independent experiments.

293/RGP cells were co-transfected with the vectors expressing the NefG3C-GFP fusion product and the p75 human Nerve Growth factor receptor truncated in its intracytoplasmic domain (ΔNGFr). Indeed, the expression of this marker was useful in the first selection steps, when the cell sorter-based selection led the cells to an unacceptable cell mortality. In this respect, after two anti-NGFr selections followed by two GFP-based cell sorting, we recovered a cell population (18-4s cells) more than 95% positive for the NefG3C-GFP expression (Figure 2A). Of note, at the FACs analysis 18-4s cells actually appeared as two distinct sub-populations in terms of the expression of both NefG3C-GFP and ΔNGFr. 18-4s cell grew with a similar duplication time to that of the parental cells (not shown), but tended to float much more easily. The levels of NefG3C-GFP remained stable during two months period in culture, but progressively declining thereafter (not shown). This appeared to be the consequence of the silencing rather than the loss of the NefG3C-GFP sequences, since treatment with sodium butyrate restored a GFP FACs profile similar to early-passage cells (not shown).

We then sought to establish the best experimental conditions for the induction of the expression of HIV-1 products in 18-4s cells. Following the methods previously described for the 293/RGP cells [23], we treated 18-4s cells with 5 mM sodium butyrate and/or PonA 2 μM, and monitored the expression of HIV-1 CAp24 and NefG3C-GFP by FACs two and three days later. Specifically, 293/RGP and 18-4s cells were treated with: i) PonA alone; ii) both PonA and sodium butyrate for one day and, thereafter, with PonA alone, or iii) with PonA and sodium butyrate. As shown in Figure 2B, the treatment with PonA alone resulted in inefficient expression of Gag products in 18-4s cells, while more than 50% of the parental 293/RGP cells was induced to express Gag products. The addition of sodium butyrate was mandatory for significant Gag induction in 18-4s cells. In fact, a pulse of sodium butyrate for 24 h resulted in 60% of the cells expressing Gag whereas its continuous presence for the 48 h induction period further increased the percentage of positive cells to 77%. Additionally, the expression of NefG3C-GFP was increased 3–4 fold. Sodium butyrate treatment increased also the percentage of Gag positive 293/RGP cells from 57% to more than 80%, and improved the relative mean fluorescence intensity by 2–2.5 fold. Of note, in 18-4s cells the treatment with sodium butyrate alone led to an increase of the NefG3C-GFP expression similar to that induced also in the presence of PonA (not shown). No remarkable differences in terms of expression of both CAp24 and NefG3C-GFP were observed between the cells harvested at the day 2 and at the day 3 (not shown). At this time, however, the cell cultures suffered of some cell mortality (not shown).

Finally, the intracellular localization of NefG3C-GFP we analyzed by fluorescence microscope seemed qualitatively similar to that already described for wt Nef [25] in the presence, however, of a stronger localization at the cell membrane (not shown).

### Dose-response of the PonA induced production of VLPs from 18-4s cells

**Figure 3:**
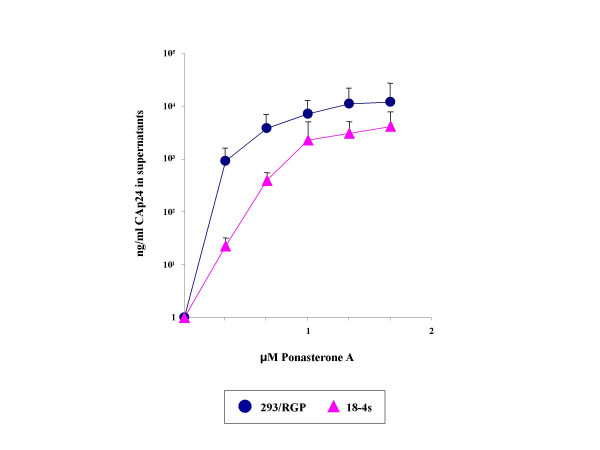
**The efficiency of VLP production from 18-4s cells depends on the PonA concentration**. PonA dose-response of the VLP production from 18-4s cells as compared with the parental 293/RGP cells. The cells were cultivated for 3 days in the presence of 5 mM sodium butyrate together with increasing doses of PonA. Supernatants were clarified and the VLP contents measured as concentrations of HIV-1 CAp24. The mean values ± s.e. from the data of three independent experiments are reported.

Clearly, the most relevant feature of 18-4s cells was expected to be the ability to produce fluorescent VLPs. In this regard, the VLP production efficiency was assayed by inducing 18-4s cells with increasing concentrations of PonA in the presence of the optimal doses of sodium butyrate (i.e., 5 mM) that was mandatory for the expression of HIV-1 CAp24 and related products. As for 293/RGP, 18-4s cells produced VLPs at concentrations that increased with the doses of PonA up to 2 μM (Figure 3). Higher doses of PonA did not further increase the amounts of produced VLPs (not shown). At the highest concentrations of PonA, 18-4s cells produced amounts of VLPs equivalent to up to 4 μg/ml. However, these levels were still 2–3 fold lower compared with those from the parental cells. This could be the consequence of the incorporation of the high number of NefG3C-GFP molecules that may in some way decrease the efficiency of the HIV assembling/budding process.

### Characterization of NefG3C-GFP VLPs

**Figure 4:**
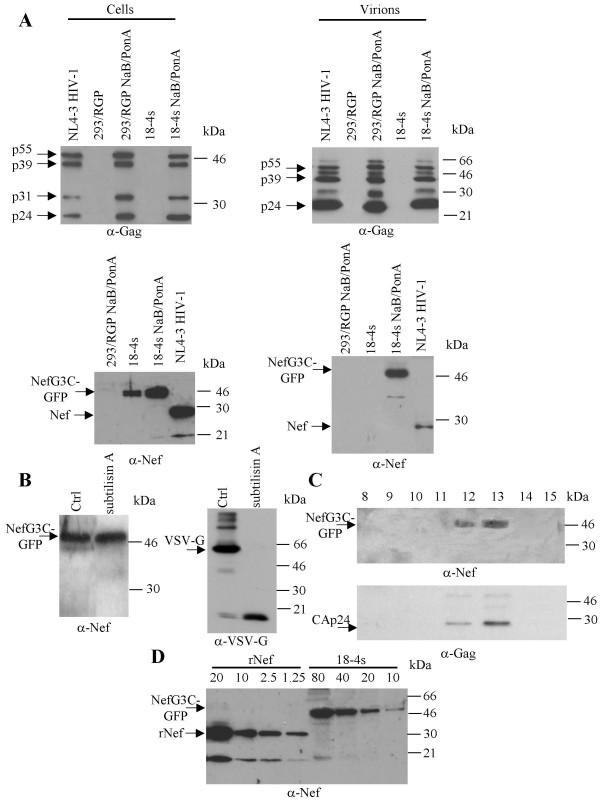
**Molecular analysis of the HIV-1 related products in 18-4s cells and VLPs**. (A). Western blot analysis for the cell expression and VLP incorporation of both HIV-1 Gag-related products and NefG3C-GFP in control and/or induced 18-4s and parental 293/RGP cells. As control, both cell and viral lysates from HIV-1 pNL4-3 transfected 293T cells were used. Fifty μg of total proteins from each cell lysate or 50 ng of VLPs were assayed using a 1:1000 dilution of pooled strongly HIV-1 positive human sera, or a 1:1000 dilution of the ARP444 anti-Nef sheep antiserum. NaB: sodium butyrate. (B) Western blot analysis of subtilisin A treated 18-4s VLPs. Hundred ng of (VSV-G) NefG3C-GFP VLPs were treated with 10 μg of subtilisin A or with an equal amount of bovine serum albumine (Ctrl) and, after 2 hours of incubation at 37°C, the reaction was stopped with phenylmethylsulfonyl fluoride, and the samples analyzed for both NefG3C-GFP and VSV-G contents by Western blot using the ARP444 anti-Nef polyclonal Abs or anti-VSV-G polyclonal Abs recognizing the VSV-G intracytoplasmic tail. The lowest signals overlaps the front-dye of the SDS-PAGE where degraded protein products are expected to accumulate. (C) Anti-Nef and anti-CAp24 Western blot analysis of relevant fractions from an iodixanol gradient loaded with 18-4s VLPs. Fifteen ml of supernatant from induced 18-4s cells containing about 50 μg of VLPs were concentrated by ultracentrifugation on a 20% sucrose cushion, and loaded on a 6 to 35% discontinuous iodixanol gradient. The Western blot analysis of the eight most relevant fractions out of the fifteen harvested is reported. (D) Quantitation of NefG3C-GFP virion incorporation. Two-fold serial dilutions of 18-4s VLPs were analyzed by Western blot for the presence of NefG3C-GFP as compared with serial dilutions of rNef. The nanograms of rNef and 18-4s VLPs, the latter measured by quantitative anti-CAp24 ELISA, are indicated on the top. Data are representative of 8 (A), 3 (B), and 2 (D) independent experiments. For all panels, the migration of major viral products are indicated on the left side, whereas the molecular marker sizes are reported on the right.

Next, we molecularly characterized the VLPs released by 18-4s cells. The Western blot analysis for the cell associated HIV-1 Gag-related products did not show significant differences compared with the parental cells, while the anti-Nef Western blot analysis, consistently with the here above reported results, revealed an induction-dependent apparent increase of the NefG3C-GFP expression (Figure 4A). Concerning the VLP molecular composition, no significant differences were detected in the contents of Gag-related products between HIV-1 and VLPs from 293/RGP or 18-4s cells. On the other hand, and of a major relevance, remarkable amounts of NefG3C-GFP molecules appeared to be associated with the VLPs from 18-4s cells, as also confirmed by the anti-GFP Western blot analysis (not shown). However, in order to ensure that the NefG3C-GFP molecules we detected were indeed incorporated and not just associated with their surface, we additionally performed Western blot analysis after a proteolytic treatment of the VLPs. Thus, VLPs from 18-4s which had been pseudotyped with the glycoprotein receptor of the Vesicular Stomatitis virus (VSV-G) were treated with the subtilisin A protease and analyzed for the presence of both NefG3C-GFP and VSV-G. Such a treatment was expected to degrade the viral surface proteins as well as any products non specifically associated with the viral envelope, but should not affect genuinely incorporated proteins. As shown in Figure 4B, while the NefG3C-GFP related signal appeared substantially unaffected by the subtilisin A treatment, the VSV-G receptor resulted efficiently degraded. To further support the idea that the Nef-related signals we detected by Western blot analysis indeed originated from virion incorporated NefG3C-GFP molecules, VLPs were purified through a iodixanol gradient, and the recovered fractions analyzed by Western blot (Figure 4C). The co-sedimentation of NefG3C-GFP and CAp24 strengthens the conclusion that NefG3C-GFP molecules are indeed incorporated in VLPs.

Furthermore, we were interested in estimating the number of molecules incorporated in the VLPs released from induced 18-4s cells. To this end, we compared the intensity of the signals from decreasing amounts of recombinant (r) Nef with those from decreasing amounts of VLPs whose contents in CAp24 were preventively and rigorously established by quantitative ELISA (Figure 4D). On the basis of the densitometric analysis carried out on the Western blot films (not shown), we estimated that 20 ng of 18-4s VLPs contain approximately 1.25 ng of NefG3C-GFP molecules. Considering the purity of rNef preparation (i.e., 95%), the differences in the molecular weight between CAp24 and NefG3C-GFP, and that it was reported that HIV-1 particles incorporate approximately 5,000 molecules of CAp24 per virion [26], we calculated the presence of about 150 NefG3C-GFP molecules per VLP. Such an estimation cannot take account of possible differences in the antibody recognition between NefG3C-GFP and wt rNef. These, however, are not expected to be of a major relevance since the anti-Nef polyclonal Abs preparation we employed was recovered using a full-length wt rNef derivative as immunogen.

**Figure 5:**
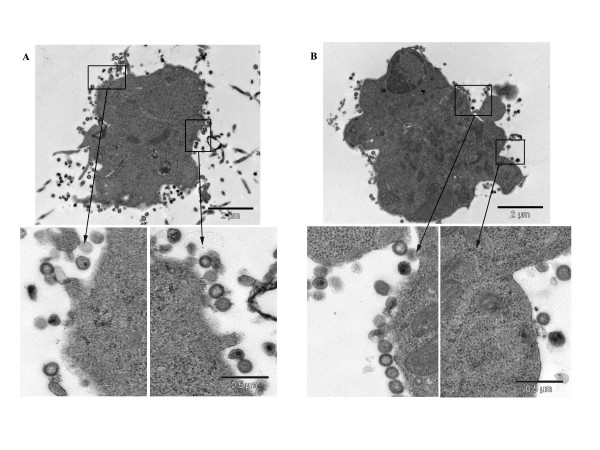
**Transmission electron microscope analysis of VLPs released by 18-4s cells**. Thin section transmission electron microscope analysis of 293/RGP (A) and 18-4s cells (B) two days after induction. Relevant morphologic details regarding the produced viral particles were enlarged in the respective inserts. Bars mark 2 μM and 0.5 μM in the low- and high magnification images. respectively.

Finally, we analyzed the morphology of the VLPs released by induced 18-4s cells by electron microscope as compared with VLPs from parental 293/RGP cells. Clearly, 18-4s cells released VLPs with a morphology very similar to that of the VLPs from parental cells (Figure 5A–B), thus suggesting that the NefG3C-GFP incorporation did not grossly influence the VLP morphology significantly. Both VLP populations appeared composed of immature and mature viral particles, the latter bearing the typical electron dense conical cone structures. Of note, in both cell cultures, the presence of immature VLPs raised significantly with the cell passages (not shown)

In conclusion, our data indicate that, upon induction, 18-4s cells produce HIV-1 VLPs whose morphology was not influenced by the high levels of incorporated NefG3C-GFP molecules.

### Cytofluorimetric detection of fluorescent NefG3C-GFP VLPs

**Figure 6:**
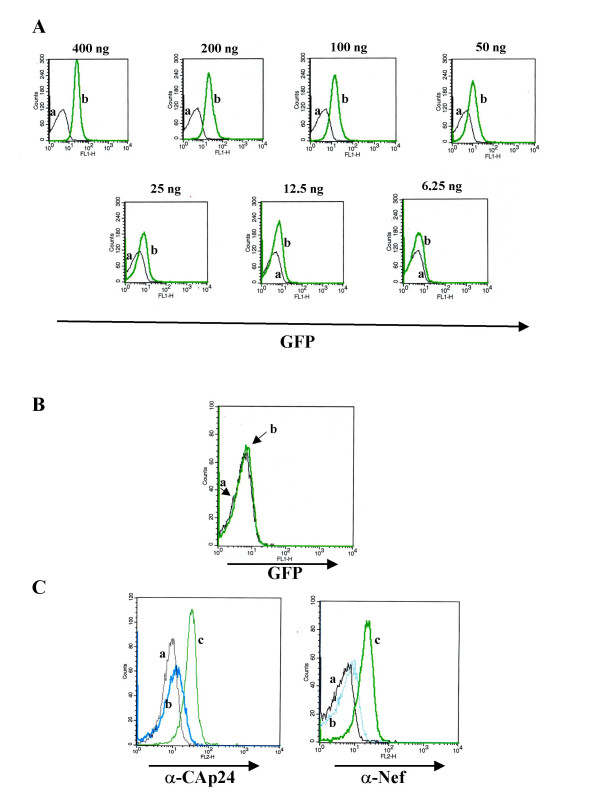
**Cytofluorimetric analysis of VLPs released by 18-4s cells**. (A) Hundred microliters of supernatants from induced 18-4s cells were clarified, adjusted to 400 ng of CAp24 contents, two-fold serially diluted in complete medium, incubated with aldehyde latex beads, and finally analyzed by FACs (slopes b). As control, supernatants from 293/RGP cells adjusted for equal VLP contents were used (slopes a). (B) The supernatants from induced 18-4s (slope b) and, as control, from 293/RGP cells (slope a) were cleared from a total of 400 ng of VLPs by ultracentrifugation, and then analyzed by FACs upon incubation with aldehyde latex beads. (C) Hundred ng of 18-4s VLPs were incubated with aldehyde latex beads and labeled with either KC57 PE-conjugated anti-CAp24 (on the left) or anti-Nef mAbs # 6.2 (on the right) and, in a second step, with PE-conjugated goat anti-mouse IgG. Histograms refer to the FACs analysis of aldehyde latex beads incubated with supernatants of 18-4s cells either non induced (a) or induced but in the absence (b) or presence (c) of the permeabilization step before the Ab labeling.

Next, we developed a rapid and sensitive assay for the detection of the VLP-associated fluorescence based on the ability of aldehyde latex beads to specifically bind lipid enveloped micro- and nanoparticles. Similar tests were previously applied to the analysis of exosomes [27], and here we originally provide evidence that such a method can be fruitfully exploited for the analysis of VLPs. In detail, we incubated clarified supernatants from induced 18-4s cells with the aldehyde latex beads at r.t. that, 1–2 hours later, were extensively washed and analyzed by FACs. The representative results reported in Figure 6A demonstrate that this assay can detect as few as 25 ng of 18-4s VLPs measured as CAp24 contents. The possible presence of free NefG3C-GFP molecules did not interfere with the FACs measurements since no fluorescence increase was found associated to the beads incubated with the supernatants from induced 18-4s cells previously cleared from VLPs by ultracentrifugation as compared with the control supernatants (Figure 6B). In addition, the actual binding of VLPs to the beads was confirmed by the specific labeling of either virion-associated CAp24 or Nef with monoclonal Abs we observed only upon a permeabilization step (Figure 6C).

**Figure 7:**
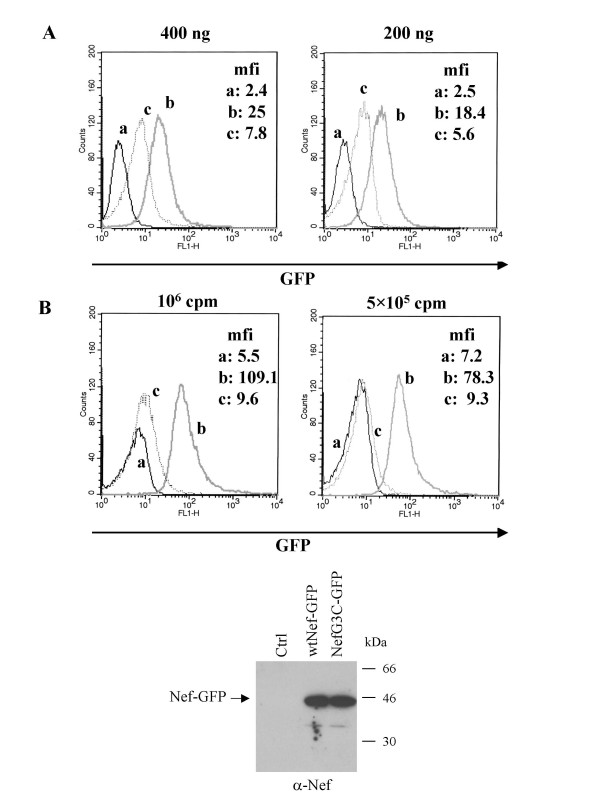
**Comparative analysis of the GFP-related fluorescence associated with NefG3C-GFP and GFP-Vpr VLPs**. (A) The indicated amounts of NefG3C-GFP (b) and GFP-Vpr (c) VLPs purified from the supernatants of transfected 293T cells were incubated with aldehyde latex beads and then analyzed by FACs. As control, the analysis of beads incubated with equivalent amounts of void VLPs (a) was included. The mean fluorescence values for each slope are indicated in the respective panels. The results are representative of data obtained from two independent experiments. (B) Analysis of the GFP-related fluorescence associated with MLV-based VLPs produced in cells expressing NefG3C-GFP (b) or wt Nef-GFP (c). 293T cells were co-transfected with a MLV packaging construct and a vector expressing wt Nef-GFP or NefG3C-GFP. VLPs released in the supernatants were purified, and the relative amounts quantified through the Mn^++ ^dependent reverse transcriptase assay. Amounts of VLPs equivalent to 10^6 ^or 5 × 10^5 ^cpm were incubated with aldehyde latex beads and analyzed by FACs as compared to equal amounts of void MLV VLPs (a). The mean fluorescence values for each slope are indicated in the respective panels. The results are representative of data obtained from two independent experiments.

We exploited such VLP analytical method to compare the fluorescence associated with NefG3C-GFP VLPs with that of VLPs incorporating GFP-Vpr. To this aim, 293T cells were co-transfected with a HIV-1 packaging vector together with the vector expressing the respective GFP-related product, and the released VLPs purified on a 20% sucrose cushion. Then, equivalent amounts of VLPs, as measured by a quantitative anti-CAp24 ELISA, were incubated with aldehyde latex beads, and the fluorescence evaluated by FACs. As shown in Figure 7A, beads binding NefG3C-GFP VLPs showed more than 3-fold increased fluorescence as compared with the GFP-Vpr VLPs. This was not the result of different transfection efficiencies in the respective producer cells, where the GFP-related products appeared equally expressed (not shown), but is consistent with the VLP Western blot analysis depicted in Figure 1B.

Next, to assess whether the NefG3C-GFP system could be applied also to other retroviruses, we evaluated the levels of incorporation of NefG3C-GFP molecules in Moloney Leukemia Virus (MLV) particles, that were shown to efficiently incorporate HIV-1 Nef [28]. To this aim, we compared the fluorescence associated to VLPs produced in the presence of wt Nef-GFP with that of NefG3C-GFP. 293T cells were co-transfected with the vector expressing the respective GFP-related product together with a Gag-Pol MLV packaging construct, and equivalent amounts of purified VLPs, as measured by the Mn^++ ^dependent reverse transcriptase assay, were incubated with aldehyde latex beads. The FACs analysis (Figure 7B) clearly showed low levels of fluorescence associated with wt Nef-GFP MLV VLPs, while strong GFP-related signals arose from the beads binding the NefG3C-GFP MLV VLPs. This result represents the proof-of-principle that the NefG3C-GFP approach could be of utility also for non HIV-1 retroviral systems.

### Pseudotyped 18-4s VLPs efficiently deliver NefG3C-GFP molecules in target cells

**Figure 8:**
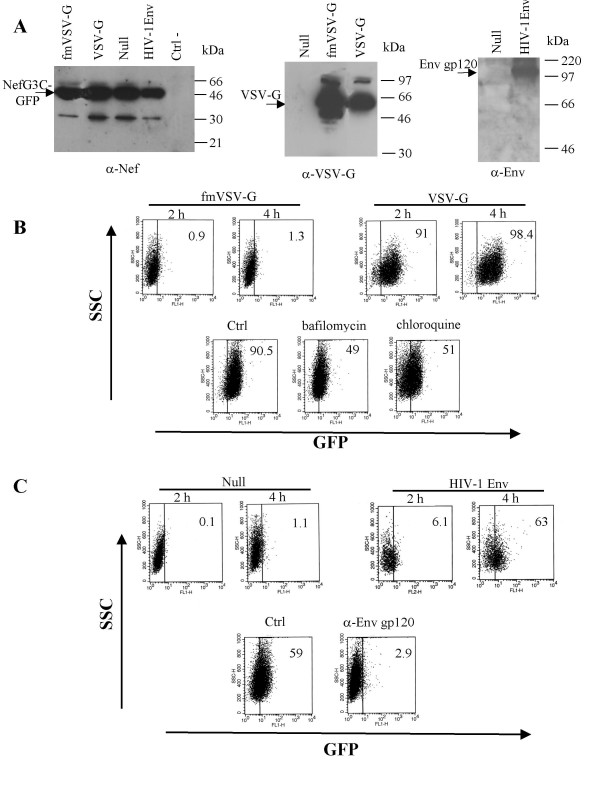
**Efficient delivery of the NefG3C-GFP-related fluorescence by pseudotyped 18-4s VLPs**. (A) Western blot analysis of NefG3C-GFP pseudotyped VLPs. Hundred ng of differently pseudotyped VLPs were analyzed by Western blot for the presence of NefG3C-GFP and of the respective receptors. The migration of major viral products is indicated on the left side, whereas the molecular marker sizes are reported on the right. (B) FACs analysis of CEMss cells 2 and 4 hours after the challenge with 250 ng/10^5 ^cells of NefG3C-GFP VLPs pseudotyped with VSV-G or, as control, with its mutant defective for the fusion activity (fm). As control, in a separate experiment the same amounts of (VSV-G) VLPs were used to challenge cells untreated (Ctrl) or pre-treated for 2 hour at 37°C with either 100 nM bafilomycin A1 or 50 μM chloroquine. Cells were analyzed 2 hours after the challenge. Bars were drawn at the highest fluorescence levels of untreated cells. Percentages of GFP positive cells are reported in the respective plots. (C) FACs analysis of CEMss cells 2 and 4 hours after the challenge with 1 μg/10^5 ^cells of NefG3C-GFP VLPs pseudotyped with X4-tropic HIV-1 Env or, as control, without receptors ("Null"). As control, in a separate experiment the same amounts of HIV-1 Env VLPs were incubated for 1 hour at 4°C with the 17b anti-Env gp120 neutralizing mAb, or with an isotype matched irrelevant mAb (Ctrl) before the cell challenge. Cells were analyzed 4 hours after the challenge. Bars were drawn at the highest fluorescence levels of untreated cells. Percentages of GFP positive cells are reported in the respective plots.

Finally, we were interested in establishing whether and how efficiently 18-4s VLPs can fluorescently mark the target cells upon viral entry. To this aim, 18-4s VLPs pseudotyped with either the VSV-G (in its wt form or with a mutant defective for the fusion activity) [29] or the HIV-1 X4 Env receptors were produced by transfecting 18-4s cells with the appropriate expression vectors and, 6 to 8 hours later, treating the cells with PonA and sodium butyrate. Of note, the liposome-mediated transfection worked efficiently in 18-4s as well as in 293/RGP parental cells, ranging the expression of transfected receptors from 60 to 95%, as measured by FACs (not shown). Thus, 18-4s cells remained a good recipient for the expression of ectopic sequences. VLP preparations were preventively characterized in terms of their contents of both NefG3C-GFP molecules and the respective receptors (Figure 8A). Next, we assessed that 250 ng/10^5 ^cells of VLPs pseudotyped with the wt but not with the fusion-mutant VSV-G rendered the majority of challenged cells fluorescent at the FACs analysis as early as 2 hours after the VLP treatment. (Figure 8B). In addition, the pre-treatment of target cells with either bafilomycin A1 or chloroquine, i.e. two drugs inhibiting the VSV-G mediated endocytic fusion by raising the endosomal/lysosomal pH, led to a clear reduction of the fluorescence of the challenged cells (Figure 8B). We interpret the residual cell-associated fluorescence as the product of the accumulation of fluorescent VLPs in not functional endosomes/lysosomes rather than of a suboptimal cell response to the inhibitors. In fact, similar outcomes were obtained also by raising the drug concentrations up to 5-fold (not shown).

Conversely, and as expected, the viral entry driven by HIV-1 Env appeared significantly less efficient, considering that 1 μg/10^5 ^cells of HIV-1 Env pseudotyped VLPs rendered fluorescent about 40% of target cells that also internalized lower amounts of NefG3C-GFP, as indicated by the lower mean fluorescence intensity (Figure 8C). The idea that the fluorescence detected in target cells indeed relied on authentic viral fusion events was strengthen by the inhibition we observed upon the pre-incubation of HIV-1 Env VLPs with either the 17b anti-Env gp 120 (Figure 8C), or the 2F5 anti-Env gp41 neutralizing mAbs (not shown).

**Figure 9:**
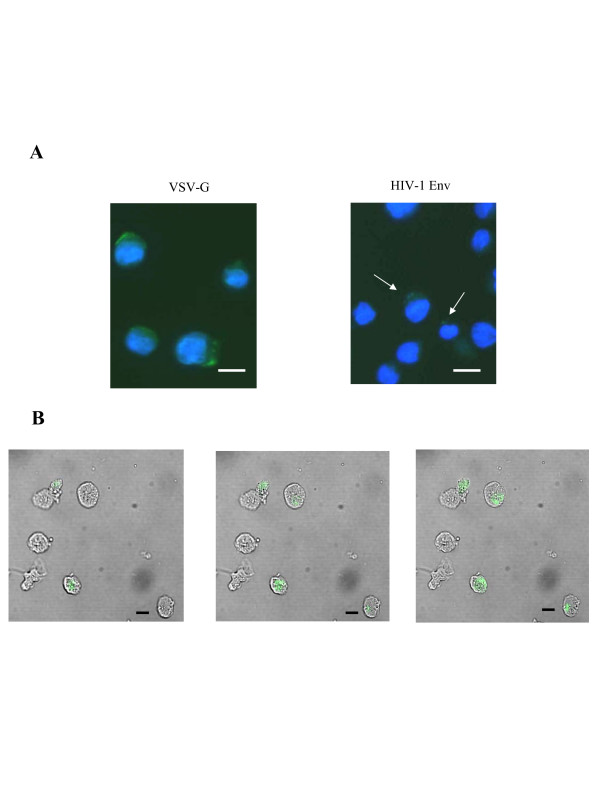
**Fluorescence and confocal microscope analysis of VLP challenged cells**. (A) Fluorescence microscope analysis of CEMss cells 4 hours after the challenge with VSV-G (on the left) or X4 HIV-1 Env (on the right) pseudotyped NefG3C-GFP VLPs after DAPI labeling. The bars mark 10 μM (B) Confocal microscope analysis of CEMss cells 4 hours after the challenge with (VSV-G) NefG3C-GFP VLPs. The same field was scanned at three section plans. The images are the result of the computer-assisted analysis carried out by overlapping the images obtained with visible and fluorescence lights. The bars mark 10 μM.

The analysis at the fluorescence microscope of the target cells (Figure 9A) showed a strong fluorescence associated with (VSV-G) NefG3C-GFP VLP challenged cells, while the fluorescence levels appeared significantly fainter in the cells challenged with the X4 Env pseudotyped VLPs. Finally, the confocal microscope analysis formally confirmed that the fluorescence associated with the target cells originated from an authentic cell internalization of fluorescent VLPs pseudotyped with VSV-G (Figure 9B), or with HIV-1 Env receptors (not shown). Besides the above described challenges carried out with VLPs lacking functional receptors, such results were validated also by the lack of fluorescence we observed in cells challenged with either VSV-G or X4 Env pseudotyped VLPs and incubated at 4°C (not shown). Finally, the results obtained in CEMss cells were also reproduced in U937 cells, primary CD4+ lymphocytes, or human primary monocyte-derived macrophages (not shown).

Taken together, our data support the idea that the 18-4s cells could be a useful reagent for the study of early events correlating with the viral entry mediated also by heterologous virus receptors.

## Discussion

To the best of our knowledge, this is the first report describing the isolation of a stable cell population releasing fluorescent HIV-1 based VLPs. The biological features of 18-4s cells do not diverge significantly from those of the 293/RGP parental cells [23], except for a more stringent requirement of sodium butyrate for the VLP production. Sodium butyrate is known acting on methylated promoter sequences and/or on surrounding heterochromatinic DNA, ultimately leading to the re-activation of silenced genes. This is thought occurring through the hyperacetylation of histone and non-histone nuclear proteins [30,31]. We observed that, in the one hand, the parental 293/RPG cells significantly decreased the levels of VLP release with passages. This was possibly due to a progressive transcriptional silencing of HIV-1 regulatory and/or structural genes, and was efficiently reverted by sodium butyrate. This treatment, on the other hand, was absolutely required for the release of VLPs from 18-4s cells, likely due to the silencing of HIV-1 genes already operative at the time of the cell isolation.

The FACs analysis revealed that 18-4s cells are composed by two clearly distinguishable sub-populations in terms of the expression of both NefG3C-GFP and ΔNGFr. In particular, on the basis of the mean fluorescent intensities we measured by FACs, the bright sub-population seemed to accumulate both products about 5-fold more efficiently than the dull one. Concerning the most relevant expression of NefG3C-GFP, we noticed that with passages, the bright sub-population tended to reduce, meanwhile appearing a detectable fraction of GFP negative cells. Again, this is likely the consequence of the silencing of the NefG3C-GFP sequences rather than to their loss since also in this case the treatment with sodium butyrate resulted in the restoration of the original GFP profile (not shown). We have no obvious explanations for the mechanisms underlying the production of the two sharply distinct 18-4s cell sub-populations, even if it may be conceivable that this reflects the differences in the number and/or sites of the integration of the plasmid vectors. In addition, the consequent protein overproduction might be associated with an impaired protein degradation efficiency, thus resulting in steady-state protein levels overriding the real differences in the numbers of integrated vectors.

The 2–3 fold decreased levels of VLP release from the 18-4s cell line compared with the parental cells appeared consistent with the reduced viral production we observed in transient transfection experiment (not shown), and was possibly due to some steric hindrance occurring during the assembling/release process as the consequence of the high levels of NefG3C-GFP incorporation. Of note, the data from EM analysis showed that this did not lead to significant morphologic alterations of VLPs that in both cases appeared as both mature and immature particles. In this regard, we cannot formally exclude that the significant presence of immature viral particles depended at least in part on a slow kinetic of maturation rendering more difficult the detection of mature particles through the electron microscope observation of ultra-thin cell sections we performed.

The analyses we carried out by FACs and by fluorescence and confocal microscopes consistently indicated that the cells targeted by pseudotyped 18-4s VLPs become fluorescent as early as 2 hours after the challenge. Generally speaking, the great part of HIV-1 particles undergo cell internalization through a receptor independent endocytosis leading to lysosome degradation [10,32], whose inhibition, in fact, increases HIV infection [33,34]. In the analysis of the VLP entry we performed, it is worthy of note that the 18-4s VLPs pseudotyped with the fusion defective VSV-G or with "null" VLPs did not induce a significant increase of the cell fluorescence compared with the mock challenged cells. These results, together with those obtained with chemical inhibitors or neutralizing antibodies, strongly suggests that the cell fluorescence we detected basically relied on the completion of the virus fusion events with the release into the cytoplasm of the fluorescent molecules, a conclusion also supported by our results from the confocal microscope analysis.

We feel the relevance of the 18-4s cells stands in two main fields of application. The first could be the study of the mechanisms and dynamic of viral entry, and the second regards the screening of antiviral compounds targeting the late HIV replication events. In particular, the possibility to pseudotype HIV-1 VLPs with a wide array of viral receptors could allow gaining more insights on the not yet fully elucidated mechanism of entry of viruses of different species. This is for instance the case of Human hepatitis C virus, whose cell receptor/co-receptors have still to be unambiguously identified (for a review, see [35]). In this regard, the cell fluorescence generated by the VLP entry would be a more stringent marker of viral entry compared with the in use systems relying on the lentivirus-mediated transduction of gene markers whose expression can be restricted by host cell factors. On the other hand, the rather simple protocol for the detection of fluorescent VLPs by FACs we developed renders 18-4s cells a both safe and powerful tool for large-scale screenings of new anti-HIV compounds electively targeted to the assembly/release processes. Thus, 18-4s cells are anticipated to be a useful reagent for multiple purposes that will be freely available upon request.

Furthermore, from the point of view of the basic virology, the possibility to analyze viral particles by FACs as here described opens the way towards new rapid and accurate approaches in the investigation on the molecular composition of enveloped viruses in terms of both viral and virus-associated cell products.

Finally, on the basis of the results we obtained with the MLV-based VLPs, we expect that the NefG3C-GFP approach could be applied also to different retroviral systems close to HIV-1(e.g., HIV-2, SIV) and MLV.

## Conclusion

Fluorescent VLPs produced by the 18-4s cell line can be considered a novel reagent useful for studies of virus entry also in fields other than HIV. In addition, by exploiting a FACs-based assay for the detection of the VLP fluorescence we here originally describe, 18-4s cells would be of a great utility for screening new generations of antiviral compounds targeting late events of HIV-1 replication.

## Methods

### Molecular constructs, cell lines and cultures

The vector expressing the NefG3C-GFP mutant was generated from the pcNefsg25GFP vector using the quickchange kit (Stratagene, La Jolla, CA), and the mutation was checked by sequencing. 18-4s cells were recovered by the liposome (Lipofectamine-2000, Invitrogen, Carlsbad, CA)-mediated transfection of the HIV-1 packaging 293/Rev-Gag-Pol cells [23] with vectors expressing NefG3C-GFP and the human Nerve Growth factor receptor truncated in its intracytoplasmic domain [36]. 293T, 293/Rev-Gag-Pol, and 18-4s cells were grown in Dulbecco's modified Eagle's medium supplemented with 10% decomplemented Fetal Calf Serum (dFCS). CEMss and U937 cells were cultivated in RPMI supplemented with 10% dFCS. Human primary lymphocytes were isolated from peripheral blood mononucleated cells (PBMC) isolated from 20–40 year-old healthy male blood donors. Lymphocytes were selected from PBMC using an immunomagnetic-based selection kit from Miltenyi Bioytec (Auburn, CA). PBLs were activated with 2 μg/ml phytohemagglutinin (Sigma-Aldrich, Milan, Italy), and cultivated in RPMI containing 20% of dFCS in the presence of 100 U/ml of recombinant human IL-2 (Roche, Nutley, NJ). Monocytes were isolated by 1 h adherence of PBMC, followed by immunomagnetic depletion of non-monocytic cells carried out through a Miltenyi selection kit. Monocytes were cultivated in 48-well plates in RPMI supplemented with 20% dFCS.

### VLP and HIV-1 production, purification, titration, and challenge

Preparations of VLPs were obtained from either 293T, 293/RGP, or 18-4s cells. In the first instance, 293T cells were co-transfected by liposomes (Lipofectamine-2000, Invitrogen) with the pCMVΔR8.74 HIV-1 packaging vector [37] and the immediate-early CMV promoted vectors expressing either NefG3C-GFP, wt Nef-GFP [21], GFP-Vpr [9], or the NL4-3 wt Nef. From 48 to 72 hours later, the supernatants were harvested, clarified, and concentrated by ultracentrifugation on 20% sucrose cushion 100,000 *g*, 2 h at 4°C. Alternatively, VLP preparations were obtained from the supernatants of either 293/RGP or 18-4s cells two or three days after the induction with 5 mM sodium butyrate (Sigma-Aldrich, St. Louis, MO) and/or 2 μM of Ponasterone A (PonA) (Sigma-Aldrich). Pseudotyped VLPs from 18-4s cells were obtained by transfecting 5 to 10 μg of the vectors expressing the respective viral receptor (i.e., VSV-G or X4 Env HIV-1 from the HXB-2 isolate, both obtained from the NIH AIDS Research and Reference Reagent Program) and, 4 to 6 hours later, by inducing the cells with sodium butyrate and PonA. Twenty-four hours later, the supernatants were replaced with fresh medium containing the inducers, and VLP containing supernatants were harvested 48 and 72 hours after the induction, clarified, and purified as here above described. The purification of 18-4s VLPs was performed by a iodixanol gradient. Briefly, VLPs purified by a 20% sucrose cushion were loaded on a 6 to 35 % discontinuous gradient of Optiprep (Axis-Shield, Oslo, Norway). Samples were ultracentrifuged at 37,000 rpm at 4°C for 90 min on a SW41 rotor, and 700 μl fractions were collected from the top. These where then 1:5 diluted with PBS, pelletted, re-suspended in 100 μl, and finally 30 μl analyzed by both anti-Nef and anti-Gag Western blot.

VLP preparations were titrated by measuring the HIV-1 CAp24 contents by quantitative ELISA (Abbott, Abbott Park, Illinois), and by the reverse transcriptase assay [38]. For the cell challenges, VLPs were adsorbed by spinoculation at 150 *g *for 30 min at room temperature (r.t.). Afterwards, the cell cultures were re-fed by adding fresh medium, and incubated at 37°C. Finally, the cells were treated for 15 min with trypsin at 37°C, fixed, and analyzed by FACs. Inhibition experiments were carried out by pre-treating cells at 37°C for 2 h with either bafilomycin A1 (Sigma-Aldrich), or chloroquine (Sigma-Aldrich) for the VSV-G VLPs, or by pre-incubating HIV-1 Env VLPs for 1 hour at 4°C with the 17b or 2F5 neutralizing mAbs (200 ng for 1 μg of VLPs), both obtained from the NIH AIDS Research and Reference Reagent Program

HIV-1 NL4-3 virus preparations were recovered by transiently transfecting 293T cells with the pUc/NL4-3 infectious molecular clone [39]. HIV-1 particles recovered from the supernatants were purified and titrated as here above described.

### Western blot analysis

Both cells and purified VLP preparations were lysed in PBS, 1% Triton-X100 in the presence of anti-proteolytic agents. For the preparation of cytoplasmic extracts, whole cell lysates were centrifuged at 6,000 *g *for 10 min at 4°C, and the supernatants frozen at -80°C. Aliquots of 30 μg of total cell proteins were separated in 10% SDS-PAGE, thereby undergoing the immunoblot analysis (Western blot). rNef preparations were obtained and quantified as previously described [40]. The following mono- or polyclonal Abs served for the revelation of both VLP- and cell-associated HIV-1 products: pooled strongly reactive antisera from HIV-1 positive AIDS patients; ARP 444 sheep anti-Nef antiserum from Mark Harris, University of Leeds, Leeds, UK; polyclonal anti-VSV-G protein from Immunology Consultant Laboratories (Newberg, OR); anti-HIV-1 Env gp120 4G10 mAb from the NIH AIDS Research and Reference Reagent Program; and anti-GFP mAb from Clontech (Clontech Laboratories, Palo Alto, CA). In some experiments, VLPs were treated with subtilisin A to digest contaminating cell debris and proteins possibly non specifically adsorbed to the VLP envelope. Briefly, purified virus suspensions were supplemented with an equal volume of 2 mg of subtilisin A (Sigma-Aldrich)/ml in 40 mM Tris-HCl (pH 8.0), 2 mM CaCl_2_, and incubated 2 hours at 37°C. The digestion was stopped by addition of phenylmethylsulfonyl fluoride to 5 μg/ml.

### Fluorescence, confocal, and electron microscope analyses

For the analysis at the fluorescence microscope, 18-4s cells were placed on poly-L-Lysine (Sigma-Aldrich) coated cover glass. Cells were then fixed in 4% paraformaldehyde (Sigma-Aldrich) in PBS and quenched for 10 min with 0.1 M glycine in PBS. Cover slips were then mounted to slides using anti-fade mounting medium, after, in the cases of the 4'-6-Diamidino-2-phenylindole (DAPI, Vector Laboratories, Burlingame, CA.) labeling, cell permeabilization with 0.1% Triton-X100 (Sigma-Aldrich) in PBS. Finally, the cells were observed using a Zeiss Axioskop 2 plus fluorescence microscope. For the confocal microscope analysis, cells were fixed with 2% v/v of formaldehyde in PBS, and both phase contrast and fluorescence images were taken by an Olympus IX-81 device. For transmission electron microscopy (TEM) examination, cells were fixed in 2.5% cacodylate-buffered (0.2 M, pH 7.2) glutaraldehyde for 20 min at r.t. and post-fixed in 1% OsO_4 _in cacodylate buffer for 1 h at r.t. Specimens were then dehydrated through graded series of ethanol solutions and embedded in Agar 100 resin (Agar Aids, Cambridge, U.K.). After staining with uranyl acetate and lead citrate, ultra-thin sections were observed with a Philips 208 electron microscope at 80 kV.

### FACs analysis of cells and VLPs

For the detection of the human Nerve Growth factor receptor, 3 × 10^5^cells were incubated with a 1:100 dilution of the 20.4 mAb (ATCC, Rockville, MD, USA) or, as control, with a isotype-matched, specie-specific irrelevant mAb for 1 h at 4°C. Thereafter, cells were washed and labeled for 1 h at r.t. with a l:100 dilution of phycoerythrin (PE)-conjugated goat anti-mouse IgG. Cell populations were finally fixed with 2% formaldehyde in PBS and analyzed by FACs. For the detection of HIV-1 Gag-related products, the cells were treated with Permeafix (Ortho Diagnostic, Raritan, NJ) for 30 min at r.t., and then labeled for 1 h at r.t. with a l:100 dilution of PE-conjugated anti-CAp24 KC-57 mAb (Coulter Corp. Hialeah, FL). For the VLP detection by FACs, 5 μl of surfactant-free white aldehyde/sulfate latex beads (Invitrogen Molecular Probes, Eugene, ON) were added to 100 μl of clarified supernatants. The mixture was incubated at r.t. for 1–2 h on a rotating plate, and therefore the samples were washed once with PBS buffer. For the direct FACs analysis, the washed beads were finally resuspended in PBS-2% formaldehyde. Alternatively, the VLP-coupled beads were treated with Permeafix for 30 min at r.t., and then incubated either with a l:100 dilution of PE-conjugated of the anti-CAp24 KC-57 mAb, or with a 1:30 dilution of the anti-Nef mAb # 6.2 from the NIH AIDS Research and Reference Reagent Program.

## Authors' contributions

CM carried out the most part of the characterization of the 18-4s cells, and produced the fluorescence and confocal microscope data. PDA developed the novel FACs assay for the VLPs. AT carried out the TEM analyses. FS performed the negative staining EM observations. NS created the NefG3C-GFP vector. SS compared the virological features of 18-4s with those of parental 293-RGP cells. VB participated in the design of the characterization of the HIV-1 packaging cell lines. OS conceived the idea to generate NefG3C-based fluorescent VLPs. MF generated the 18-4s cell line, coordinated the study, and drafted the manuscript. All authors read and approved the final manuscript.
